# Colica Pictonum—Alcohol Its Exciting Cause—The Abuse of Lead

**Published:** 1868

**Authors:** Amos Sawyer

**Affiliations:** Hillsboro, Ill.


### ARTICLE VIII.

### Colica Pictonum—Alcohol its Exciting Cause—The Abuse of Lead. By Amos Sawyer, M. D., Hillsboro, Ill.

My attention was directed to this subject some years ago by the following circumstance: During the year 1851 an acquaintance, residing at Brookline, Mass., while mixing a glass of brandy, was surprised, upon adding water, to see the mixture assume a dark color. Attributing it to the liquor, he opened a new bottle, filled a clear glass, but with a like result. I would here state that the whole family had been quite unwell for some time—apparently nervous prostration. One member had a paralytic stroke; another, slight amaurosis; a third, hysteria; while all gave evidence that some mysterious agent was slowly but surely undermining their health. He, therefore, sent a sample to his family physician, (Dr. Ware) with the request to have it analyzed. It proved to be caused by *lead* contained in the water, it having been conducted through lead pipes.

As usual, the members of this family had free access to the wine-cellar, and with the second meal wine was always served. One young lady, however, seldom drank liquor of any kind, and, although *all* the others suffered more or less from colic, she was an exception to the rule, never having been troubled in that way.

This excited my curiosity, and I determined to investigate the subject. With this object in view, I have made it a practice to inquire of every painter I may meet, Have you ever known a *strictly* temperate man to suffer from lead colic? In *every* case the answer (after some deliberation, for the question seemed to take them by surprise,) was, "I have not." In but *one* instance did I receive a *prompt* reply. In this case the gentleman, who owned a large shop in Boston, had, when an apprentice some twenty years ago, observed that only those who indulged in intoxicating beverages suffered with colic; he had never drunk anything stronger than water, and so far had escaped. So fully convinced was he that it was owing to his total abstinence that he considered it his duty to warn his workmen to be temperate if they would escape. "There is no such thing as *lead*, but there is *rum* colic."

It was only last week I conversed with a very intelligent painter, recently from England. He could not recall a single instance where colic occurred in a temperate man. He says cases are far more numerous among the workmen in England than here, and suggests that it may be attributed to intoxicating beverages, particularly gin, which they think acts as a preventive.

It may be urged that colica pictonum is of rare occurrence. I admit cases seldom come under a physician's care, because they, in most cases, cease work when they feel the premonitory symptoms, and treat the diserse themselves.

In order not to be misunderstood, I will state my views succinctly. Although a strictly temperate man is equally susceptible to lead poison, only he who indulges in intoxicating beverages is in danger of, or succumbs to lead colic.

That a large portion of the inhabitants of the United States are suffering from the poisonous effects of, and that the fearful increase of neuralgia, paralysis, and a host of other nervous disorders, are consequent on the *abuse* of lead, I consider unquestionable. This may seem to be a bold assertion; but when we remember that lead in some form is introduced into every family—as pipes for pumps; painted roofs, the water from which, nine times out of ten, is conducted through painted spouts to the cistern for family use; water pails painted inside, and in universal use; old tinware, the holes in which are usually soldred with this metal by some member of the family; not to mention the different preparations of lead used as the base of all so-called cosmetics, and so on to the end of the list—I think there are few who will deny that its effect upon the general health can be otherwise but injurious.

Nor is this all. The people, as a class, are entirely ignorant of the poisonous effects of this metal. In illustration, I will cite a few instances that have from time to time come under my observation.

Case I.—Here I found the gentleman at work repairing his pump, the lead pipe having "worn away next the iron and sprung a leak." He stated that the pipe was originally ¼ of an inch thick, had been in the well for about two years, and now it had worn away for about 18 inches from the iron till it was as thin as paper. He could not account for it, as the man who sold him the pump told him that where lead and iron was in contact the acid would not affect it; and yet he was in the act of splicing on a pipe of the same metal. I explained to him its poisonous nature, and that, although his pipe was, by being in contact with iron, protected from the action of acids, he must remember the water contained an alkali, (lime) and this would cause it to be saturated with lead, and his daily dose would be far from homeopathic. He procured an iron pipe. In this case the man who sold that pump was, in every sense of the word, a criminal; for, by his very explanation, it is evident he was well acquainted with the chemical action; but because the poison acts somewhat more slowly than arsenic, he escapes his just due—State prison. The family, comprising seven members, were all subject to neuralgia; one case each paralysis and amaurosis.

Case II.—In this instance I detected the "blue line" on the gums of the father of the family; and, although aware it was not considered a characteristic sign, yet I felt warranted in announcing he was poisoned, and that lead was the agent. Further inquiry proved they used cistern water exclusively, and it was raised by a pump containing a lead pipe. He had not felt any ill effects from it, and the only evidence that my diagnosis was correct was the "blue line" and a slight weakness in the muscles of the legs. The wife suffered greatly with neuralgia, and the saturnine icterus was plainly visible. The three children seemed to be quite healthy.

Case III.—Colic: caused by eating pigs' feet that had been pickled in a new bucket. Upon examination, found the vinegar had dissolved the paint; some sulphate of baryta had settled on the bottom.

Case IV.—Colic: caused by eating poorly preserved cherries that had remained for some time in a painted vessel. The smell of paint was so disagreeahly strong I cannot conceive how any human being could have relished them. In this case the "blue line" was very plainly defined.

One word in reference to Saturnine paralysis. It has been stated, upon good authority, that the extensor muscles of the hand and forearm are usually first attacked. This has not been my experience; for, in the limited number that have come under my observation, I find it about equally divided between the muscles of the anterior femoral and the extensor muscles of the forearm. It is but just I should state that I have never seen but one case of complete paralysis, and that was of the hand. You will find, however, that old painters complain more frequently of "weakness" of the muscles of the leg than of the hand.

In conclusion, I would respectfully call the attention of the profession to this subject, hoping it will receive their careful consideration. I feel convinced that, upon investigation, it will be found to be a vein that will well repay more general investigation, and that will, in all probability, explain many cases of unexplained disease which so frequently occur in general practice, especially of that class of nervous and paralytic disorders in which, at times, there is no clue to their origin.—*St. Louis Medical Reporter.*

